# Protein Ontology (PRO): enhancing and scaling up the representation of protein entities

**DOI:** 10.1093/nar/gkw1075

**Published:** 2016-11-28

**Authors:** Darren A. Natale, Cecilia N. Arighi, Judith A. Blake, Jonathan Bona, Chuming Chen, Sheng-Chih Chen, Karen R. Christie, Julie Cowart, Peter D'Eustachio, Alexander D. Diehl, Harold J. Drabkin, William D. Duncan, Hongzhan Huang, Jia Ren, Karen Ross, Alan Ruttenberg, Veronica Shamovsky, Barry Smith, Qinghua Wang, Jian Zhang, Abdelrahman El-Sayed, Cathy H. Wu

**Affiliations:** 1 Protein Information Resource, Georgetown University Medical Center, Washington, DC 20007, USA; 2 Center for Bioinformatics and Computational Biology, University of Delaware, Newark, DE 19711, USA; 3 The Jackson Laboratory, Bar Harbor, ME 04609, USA; 4 Oral Diagnostic Sciences, University at Buffalo School of Dental Medicine, Buffalo, NY 14214, USA; 5 Roswell Park Cancer Institute, Buffalo, NY 14203, USA; 6 Department of Biochemistry & Molecular Pharmacology, NYU School of Medicine, New York, NY 10016, USA; 7 Department of Neurology, Jacobs School of Medicine and Biomedical Sciences, University at Buffalo, Buffalo, NY 14203, USA; 8 New York State Center of Excellence in Bioinformatics and Life Sciences, University at Buffalo, Buffalo, NY 14203, USA; 9 National Center for Ontological Research, University at Buffalo, Buffalo, NY 14214, USA

## Abstract

The Protein Ontology (PRO; http://purl.obolibrary.org/obo/pr) formally defines and describes taxon-specific and taxon-neutral protein-related entities in three major areas: proteins related by evolution; proteins produced from a given gene; and protein-containing complexes. PRO thus serves as a tool for referencing protein entities at any level of specificity. To enhance this ability, and to facilitate the comparison of such entities described in different resources, we developed a standardized representation of proteoforms using UniProtKB as a sequence reference and PSI-MOD as a post-translational modification reference. We illustrate its use in facilitating an alignment between PRO and Reactome protein entities. We also address issues of scalability, describing our first steps into the use of text mining to identify protein-related entities, the large-scale import of proteoform information from expert curated resources, and our ability to dynamically generate PRO terms. Web views for individual terms are now more informative about closely-related terms, including for example an interactive multiple sequence alignment. Finally, we describe recent improvement in semantic utility, with PRO now represented in OWL and as a SPARQL endpoint. These developments will further support the anticipated growth of PRO and facilitate discoverability of and allow aggregation of data relating to protein entities.

## OVERVIEW

It has long been known that the final product of a gene is inherently more complex than the gene itself (1). A single gene—even within an individual organism—can yield multiple possible specific proteoforms (the precise molecular form of a protein arising from alternative splicing events and post-translational modifications) with multiple possible functions. Proteins can also be combined in a variety of different ways within the context of multi-subunit complexes. In contrast to this increase in specificity, we can consider, more generally, cases of related sets of proteins—groups of proteins that evolved via duplication and/or speciation events of a common ancestral gene (homologs).

The Protein Ontology (PRO; see Table 1 for additional abbreviations) provides a flexible way to refer to protein entities at any such level of specificity, as generically or as precisely as needed (2). PRO organizes these entities into classes describing proteins derived from homologs (‘family level’ classes), from a single gene (‘gene level’ classes), from a single transcript (‘sequence level’ classes), or from a set of modifications (‘modification level’ classes). Each of these categories of classes are neutral with respect to taxonomy, but there are also taxon-specific versions (e.g. ‘organism-gene level’), thus allowing PRO to highlight connections and differences within and across species. In this pursuit, PRO obtains knowledge from existing resources (e.g. databases and literature), normalizes the information contained within, and provides mechanisms for information query and analysis. The normalized identifiers for protein entities at multiple levels of specificity provide appropriate targets for annotation of scientific papers.

**Table 1.** Abbreviations used

Abbreviation	Full name
GO	Gene Ontology
OBO	Open Biomedical Ontologies
OWL	Web Ontology Language
PSI-MOD	Protein Structure Initiative Modification Ontology
PTM; iPTMnet	Post-translational modification; integrated PTM network
SPARQL	SPARQL Protocol and RDF Query Language
UniProt; UniProtKB	Universal Protein Resource; UniProt KnowledgeBase
URI; URL	Uniform Resource Identifier; Uniform Resource Locator
W3C	World Wide Web Consortium

PRO is designed to facilitate the discoverability and aggregation of data relating to protein entities. Here, we describe new developments designed to further these goals, including steps taken to improve expert and automated interaction with the data, and steps taken to improve coverage and ensure scalability.

## ENHANCEMENTS OF DATA AND COVERAGE

### Standardized representation of proteoforms

**Figure 1.**
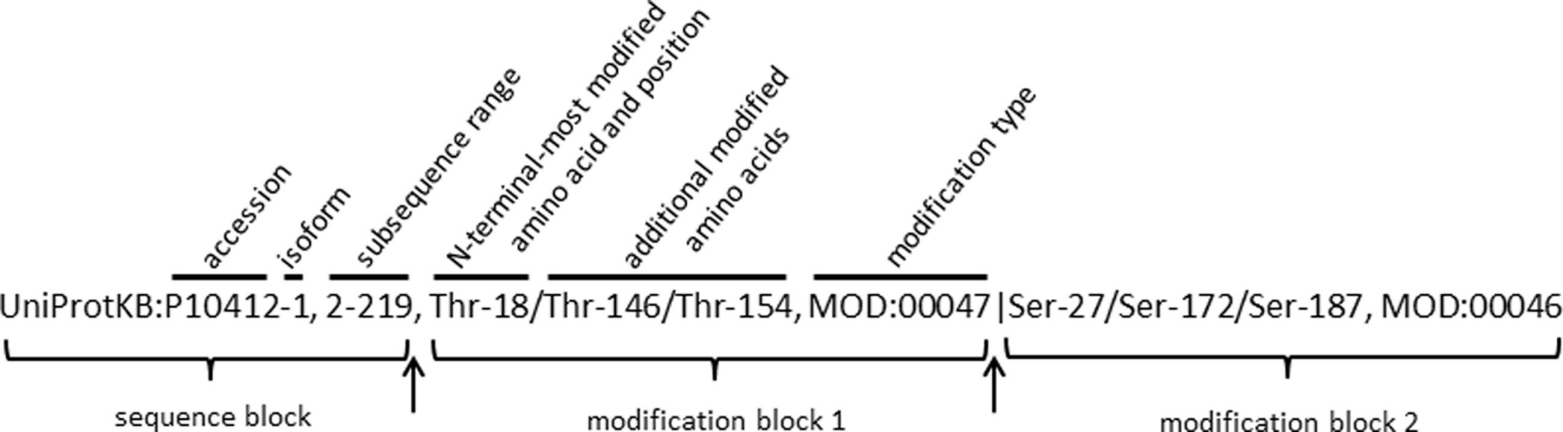
Standard representation of a proteoform. Proteoforms are represented using a standard format as annotated, consisting of a sequence block and one or more optional modification blocks. Sequence blocks consist of a UniProtKB accession with an optional isoform indicator separated by a dash, followed by a comma (first arrow) and optional subsequence range. Modification block 1, if specified, will follow a comma, and all other modification blocks will follow a pipe (second arrow). Each modification block is presented in order based on the N-terminal-most amino acid modified. Within a modification block are one or more amino acids listed by type and position, with multiples separated by slashes, followed by the PSI-MOD identifier specifying the type of modification. When an isoform is specified, N-terminal and C-terminal positions of subsequences as well as positions of modification are relative to the full length of that isoform; otherwise the numbering for the representative sequence is assumed. Only the accession is required. Missing subsequence indicates that the class encompasses either multiple species or multiple isoforms. Missing modification blocks with a subsequence indicates that the class is defined by subsequence only (such as when the only distinction is that a signal peptide has been removed).

Comparison of information from disparate resources requires the ability to discover entries that are meant to refer to identical entities. These are sometimes described in different ways. For example, Reactome (3,4) specifies the p25 form of CDK5 regulatory subunit 1 (CDK5R1(99-307), http://www.reactome.org/content/detail/R-HSA-6805262) by referring to the coordinates on the sequence given in the corresponding UniProt KnowledgeBase (UniProtKB) (5) entry (http://www.uniprot.org/uniprot/Q15078). However, since UniProtKB also assigns a ‘feature identifier’ to subsequences when a protein is cleaved in some way, it is possible to refer to that identifier instead (identifiers for subsequences are of the form ‘PRO_<*10 digits*>’ which is not to be confused with Protein Ontology identifiers, some of which are ‘PR:<*9 digits*>’ in the OBO representation and ‘PR_<*9 digits*>’ in the OWL representation). Unlike Reactome, the IntAct Complex Portal (6) specifies that same p25 form by referring to the UniProtKB feature identifier (Q15078-PRO_0000004795, cited in http://www.ebi.ac.uk/intact/complex/details/EBI-9633559). For PRO to map or import such entries, we need first to normalize these descriptions. Compounding the issue is the need to account for position-specific post-translational modifications (PTMs). We thus developed a standard syntax to indicate positions of post-translational amino acid modification on sequences or subsequences, and for specific isoforms. Our standard is similar to that used by Reactome (7) but differs in some minor details. We use UniProtKB as a source of sequence information, indicating the accession, the isoform identifier, and the subsequence range (e.g. when a proteoform has been generated via signal peptide removal or other chain cleavage). We then add the PTM information in a specific order based on the position of the modified residue nearest the N terminus, but grouped by type of modification given as a PSI-MOD (8) identifier. Additional types of modifications are then listed after a pipe character, again in sequence order, as shown in Figure 1. Having a standard representation enables us to map existing PRO terms to terms from other resources, a necessary first step to integrating the salient information in such resources into PRO.

### Addressing scalability

An ongoing problem with any curated resource is the ability to keep up with advances of scientific knowledge. This has led to increased reliance on computational means to supplement or aid expert curation. For example, PRO imports information from UniProtKB to create terms for selected species (2), and integrates information from the OMA (9) and PANTHER (10) databases to find connections between such terms. However, while such resources are well-suited for gene-level terms, we must turn to other resources to obtain increased information about specific proteoforms containing post-translational modifications. We previously described (2) the import of data from the Top-Down Proteomics Repository (11) and other resources. We now add the following data integration pipelines:

1. iPTMnet: We recently developed iPTMnet, a resource that integrates PTM information from several expert curated databases and the results of full-scale text mining of PubMed abstracts with RLIMS-P (12) that have been linked to a sequence and that have a validated phosphorylation site (Ross, K.E., Huang, H., Ren J. *et al.* 2016, in press). iPTMnet also includes phosphorylation dependent protein-protein interactions detected by a second text mining tool, eFIP (13).
  Taking advantage of iPTMnet, we implemented a fully automated workflow for creation of PRO terms for proteoforms where single phosphorylation sites are described by multiple sources (Ross, K.E., Natale, D.A., Arighi, C.N. *et al.* 2016, in press). We focused on forms phosphorylated on a single site only in our automated workflow because it is challenging, even manually, to curate proteoforms phosphorylated on combinations of multiple sites. The text mining results provided the literature evidence to distinguish between forms with single and multiple phosphorylation sites. We filtered out cases where the abstract mentioned multiple modification sites or PTM types in addition to phosphorylation (e.g. acetylation). We also filtered out cases that were already curated by PRO. After these filtering steps, ∼820 substrate-site pairs remained. These were used to automatically generate PRO entries using a template; these entries were flagged with an ‘unreviewed’ status. Annotation, including kinases and interacting partners that were extracted from iPTMnet, will be added to the terms only after expert review. The iPTMnet pipeline increased the number of organism-specific PTM proteoforms in PRO by 50%.
2. Reactome: Reactome is a pathways resource referenced by a number of projects such as Open Targets (https://www.opentargets.org/), Chemical Entities of Biological Interest (ChEBI) (14), and UniProt. Mapping between the reactants described in PRO to those in Reactome (and vice versa) will afford far greater impact than each in isolation. Using the standardized representation for proteoforms described above, we ‘translated’ proteoforms described in Reactome to the same representation by automated means. We made direct string-match comparisons to do an initial mapping (∼6300 Reactome proteins were already in PRO; most mapped to the gene level). We then created a limited set of new terms, the majority of which were proteoforms of the post-translationally processed sort (for example, amino acid modification, removed signal peptide). This resulted in a total of nearly 12 000 PRO-Reactome mappings (covering ∼60% of Reactome proteoforms). We assessed the completeness of the mapping relative to individual human pathways. For top-level Reactome pathways, PRO represents at least 50% of the participants for nearly all, with the understandable exceptions of Extracellular Matrix Organization (http://www.reactome.org/content/detail/R-HSA-1474244) and Disease (http://www.reactome.org/content/detail/R-HSA-1643685), since the first pass import skipped glycoproteins and sequence variants, among others. Neuronal System (http://www.reactome.org/content/detail/R-HSA-112316) and Transmembrane transport of small molecules (http://www.reactome.org/content/detail/R-HSA-382551) were most-highly represented, with about 90% coverage. At a more fine-grained level, PRO represents nearly 340 of the ∼1460 sub-pathways at 100% coverage and 770 sub-pathways with at least 70% coverage.
3. HIstome (15): HIstome provides a compendium of modifications to human histones, and includes the following minimum information needed to create a PRO term: UniProtKB accession (including isoform, if known), modification type (e.g. acetylation), modified amino acid and position, and experimental evidence (in the form of a PubMed reference). The data were downloaded and converted by a script to generate PRO stanzas defining each term. In a first pass, we generated 468 HIstome-evidenced PRO terms asserting that there is at least one modification on the molecule with a known position.
4. Dynamic generation of terms: A number of projects have need for PRO terms. Up to now such requests were filled fully manually. To keep up with growing demand for term requests, we have added the capability for terms of a certain type (specifically, gene-level and sequence-level terms) to be generated dynamically. We previously described our reuse of UniProtKB accessions whenever we provide an ontological representation of proteins described in that database (2). UniProtKB-derived terms can often be defined as ‘a protein that is a translation product of <*some specific gene*> in <*some specific organism*>.’ Since such definitions require only information that is available from the relevant UniProtKB entry, they can be generated by using the UniProt web service to return data on a single entry, followed by processing of that data to create a PRO term. It is important to note that while it is possible to use a persistent URL (for example, http://purl.obolibrary.org/obo/PR_E1BE92) or the specific-entry retrieval service on the main PRO web page to reference or find such terms, they will not be stored in the underlying PRO database. Thus, dynamically generated terms cannot be searched on the main page, will not be present in the downloadable PRO files or visualization of the PRO hierarchy, and will not be obtainable via SPARQL query. Nonetheless, often only a landing page is needed, and full integration of a term can be requested as described below. We plan to enhance this service with the ability to quickly request permanence of generated terms directly from the landing page, and the ability to use accessions from protein databases other than UniProtKB. We will also integrate ortholog or family information into the results.

### Addressing community needs

Community collaboration is an essential feature of PRO, as it can point to new areas of development or to specific targets for curation. Below we describe a few examples.

1. ImmPort (16): The Protein Ontology has been collaborating with the Immunology Database and Analysis Portal (ImmPort) project on the development of the ImmPort Antibody Ontology (AntiO). AntiO is an ontology that represents monoclonal antibodies, in particular those in common use in immunology research and ImmPort clinical studies. In AntiO, targets of monoclonal antibodies are identified via Protein Ontology terms, including proteoform terms for phosphorylated proteins, and protein isoforms. Over 900 antibodies are represented in AntiO, linked to their respective protein targets via expert curation. AntiO has been loaded into a triple store to allow complex queries for antibodies and antibody products based on the targeted proteoforms, antibody names, species specificity, experimental usage, and antibody product vendors, catalog numbers, and fluorochrome conjugations. We plan to use this information to make links between PRO proteoforms and the antibodies that specifically bind those proteoforms.
2. MGI: The Mouse Genome Informatics (http://www.informatics.jax.org/) group will henceforth be using Noctua (http://noctua.berkeleybop.org/), a graphical common annotation tool for gene product curation. As part of that migration, all PRO entities mapped to mouse genes will be loaded into the tool's database and made available as direct annotation objects. These mappings will be updated on a daily basis. In addition, any isoform identifiers associated with annotations imported into MGI from other sources will be converted into their PRO equivalents, and thus be normalized across all levels of specificity. This also benefits PRO in that MGI curators will be able to flag a paper for potential use in Protein Ontology curation.
3. OBO Foundry, GO Consortium, IntAct: In addition to being a part of the OBO Foundry suite of ontologies (http://www.obofoundry.org), the Protein Ontology Consortium is now an official member of the Gene Ontology Consortium (http://geneontology.org/page/go-consortium-contributors-list) and works collaboratively with several of the Model Organism Database groups and other bioinformatics resources groups as a member of the overall bioinformatics infrastructure in genetics and genomics. In this regard, PRO terms have been supplied for precise entity annotation as requested by other ontology developers (e.g. the family level term ‘CD59-like glycoprotein’ [http://purl.obolibrary.org/obo/PR_000001809] requested by the Cell Ontology [CL] (17)), and to other members of the GO Consortium (for example, the hyperoxidized form of Tpxi1 [http://purl.obolibrary.org/obo/PR_000028935] requested by PomBase (18)). GO itself uses PRO terms to define certain terms. For example, ‘response to insulin’ (http://purl.obolibrary.org/obo/GO_0032868) is logically defined as a response to a peptide hormone where that hormone is insulin (http://purl.obolibrary.org/obo/PR_000009054). Finally, PRO will serve as an intermediary connector between organism-specific complexes described in the IntAct Complex Portal and any appropriate taxon-neutral terms in GO. For example, IntAct's human muscle-type homotetramer of 6-phosphofructokinase (http://www.ebi.ac.uk/intact/complex/details/EBI-764595) would be a child of PRO's taxon-neutral version (http://purl.obolibrary.org/obo/PR_000027287), which in turn would be a child of GO's generic term for any type of 6-phosphofructokinase complex (http://purl.obolibrary.org/obo/GO_0005945).

## NEW WEB VIEWS AND RESOURCES

### Enhanced web pages for PRO terms

**Figure 2.**
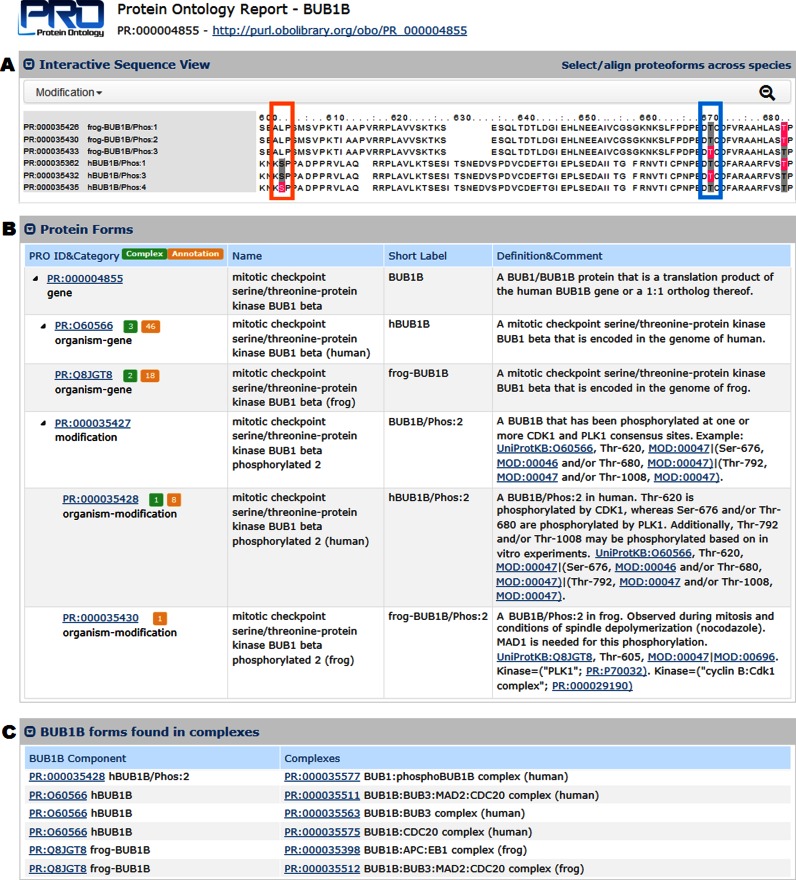
Sections of the PRO web page for the gene level protein class BUB1B (PR:000004855) and its subclasses. (**A**) Interactive Sequence View displaying a multiple sequence alignment of BUB1B proteoforms across organisms with experimentally determined phosphorylation sites highlighted in pink and potential phosphorylation sites highlighted in gray. Alignments are generated on-the-fly using MUSCLE (22) with default parameters. (**B**) Portion of the Protein Forms table displaying information about the gene level BUB1B term (PR:000004855) and several of its subclasses. (**C**) BUB1B proteoforms found in at least one complex.

We previously described a web-based comparative view for PRO terms corresponding to all proteins derived from a single gene in a single organism (2). The display contained information not only about the term itself, but also about the term's subclasses and any associated annotation. We now apply that same type of view to terms that correspond to a given gene across multiple species. Furthermore, we have enhanced the display by adding new features. To facilitate a more direct comparison between proteoform sequences and positions of post-translational modification, we now include an annotated sequence alignment. An example featuring the mitotic checkpoint protein BUB1B (PR:000004855) is shown in Figure 2. The Interactive Sequence View panel (Figure 2A) displays a multiple sequence alignment of BUB1B proteoforms across organisms. Experimentally determined modifications are highlighted in color (e.g., phosphorylation sites are pink) and potentially-modifiable sites in other sequences that align with the experimentally verified sites are highlighted in gray. For example:

- Thr-608 of human BUB1B (Figure 2A, blue rectangle at right) is phosphorylated in the proteoform hBUB1B/Phos:3 (PR:000035432) but not in the two other human BUB1B phosphorylated proteoforms shown. The aligned residue in frog BUB1B, Thr-593, is phosphorylated in the proteoform frogBUB1B/Phos:3 (PR:000035433).
- Ser-543, phosphorylated in human BUB1B (hBUB1B/Phos:4, PR:000035435; Figure 2A, orange rectangle at left), aligns with a residue not amenable to phosphorylation (Leu-532) in the frog sequence, indicating that this phosphorylation event is not conserved in frog.

Users can scroll through the sequence, zoom in/out by clicking on the magnifying glass icon, and customize which sequences are shown by clicking on the ‘Select/align proteoforms across species’ link.

The Protein Forms table—previously just an unsorted flat list—has been enhanced (Figure 2B). Terms in the table are now organized according to their positions in the PRO hierarchy, and branches of the hierarchy are expandable/collapsible so that users can focus on terms of interest. Clicking on a term's PRO identifier takes the user to the web page for that term. An orange square next to a PRO identifier indicates that the term has functional annotation, and clicking on the square will take users to the section of the Functional Annotation table showing the GO terms associated with that proteoform (not shown, but described in (2)). A green square next to a PRO ID in the Protein Forms table indicates that the proteoform is found in one or more protein complexes. Clicking on the green square will take users to a table (Figure 2C) that lists the proteoforms and their corresponding complexes.

**Figure 3.**
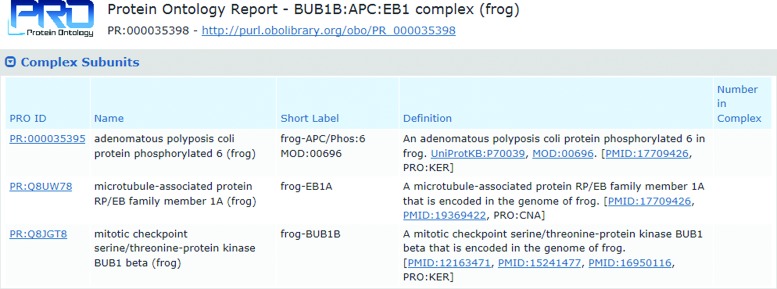
Section of the PRO web page for organism-complex term (PR:000035398) for the frog BUB1B:APC:EB1 complex showing the Table of complex subunits.

We have also developed new web pages for organism-specific complexes that provide detailed information about the complex subunits. As shown in Figure 3 for the frog BUB1B:APC:EB1 complex (PR:000035398), the Complex Subunits table lists the PRO ID, name, and definition for each of the subunits with links to the web pages for the subunit terms. A Functional Annotation table (not shown) provides any GO terms associated with the complex. From the report there are links for visualization of the hierarchy for the complex.

## PRO OWL

From its inception PRO has been distributed using the standard OBO Foundry ‘OBO’ format (http://owlcollab.github.io/oboformat/doc/GO.format.obo-1_4.html). This format has the benefit of being human readable. However, for purposes of reasoning and W3C conformity, we have added a distribution file in the OWL format (https://www.w3.org/OWL/) and are transitioning to using the OWL version as the main distributable. That format is more readily consumed by ontology query resources such as BioPortal (http://bioportal.bioontology.org/) and OntoBee (http://www.ontobee.org/).

### Pre-reasoned PRO

Two of the principal benefits of ontologies are the ability to perform internal consistency checks and to perform automated classification of terms. Each of these benefits is afforded by reasoning—an analysis of the assertions made within an ontology to make inferences about additional relationships. To use a simple example, if a protein is defined as having a phosphorylated serine, it would be classified after reasoning as a phosphoprotein, even if such was not directly stated. Reasoning can thus add additional parents to each term as appropriate. Conversely, if a protein was defined as specifically lacking any phosphorylation, yet was mistakenly classified as a phosphoprotein, reasoning would point out this inconsistency. To make such benefits available to users, we provide a version of PRO that has gone through the reasoning process. Reasoning over the latest release of PRO (v50.0) indicates there are 20 equivalencies, >80 000 new axioms and no logical inconsistencies. The reasoned version is now the default download using the links http://purl.obolibrary.org/obo/pr.obo and http://purl.obolibrary.org/obo/pr.owl.

### SPARQL endpoint

**Figure 4.**
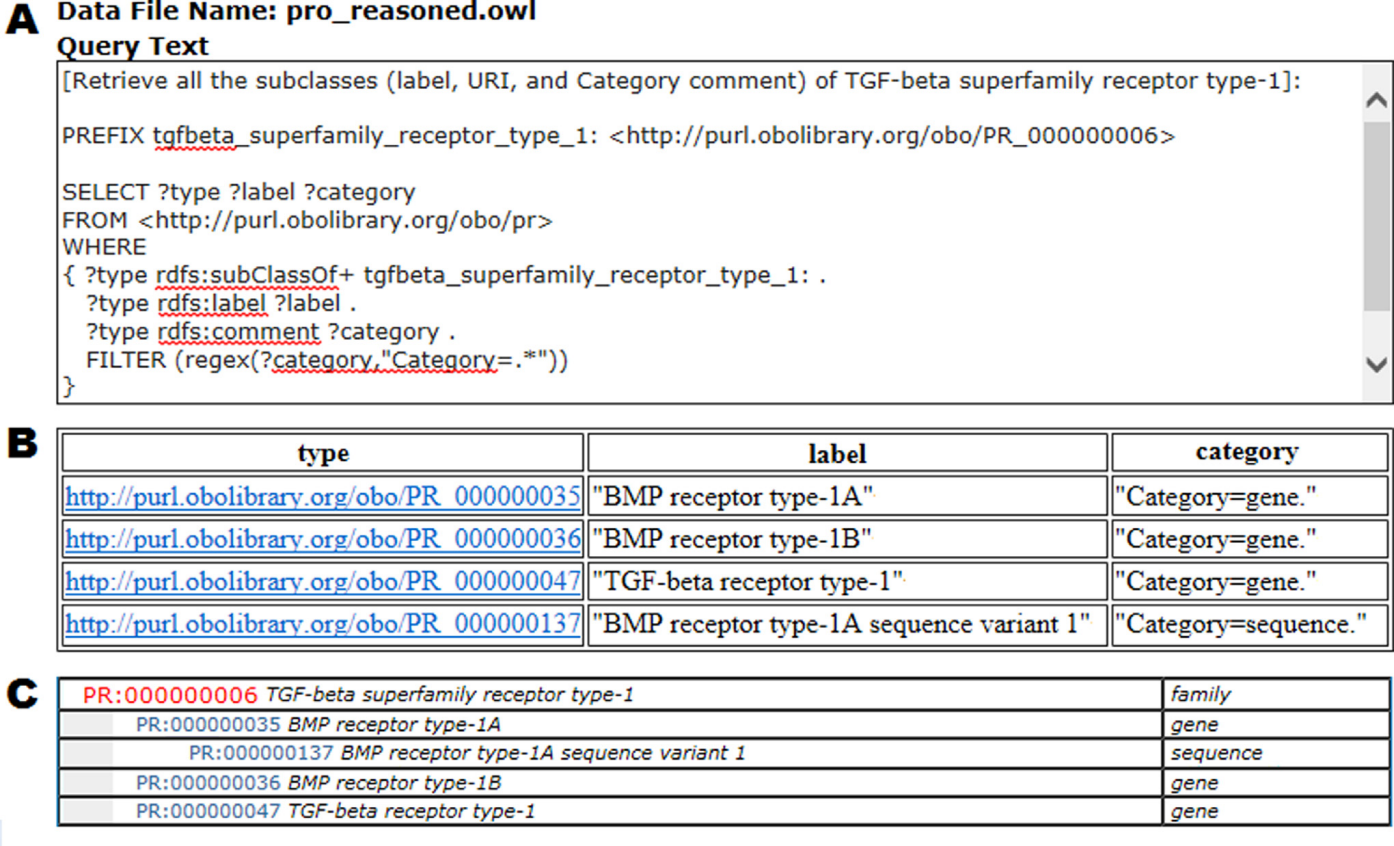
PRO SPARQL query. (**A**) A sample query designed to retrieve all the subclasses of PR:000000006, showing the term URI, label, and PRO category. (**B**) A subset of the query's 85 results. Note that for brevity the XML tag for labels and categories is not shown (the full results would have ‘∧∧http://www.w3.org/2001/XMLSchema#string’ appended to each). (**C**) The hierarchy for the subset shown in (**B**).

We have built a resource description framework (RDF) linked data repository that includes the information from the current principal PRO ontology file pro_reasoned.obo and the associated PRO annotation file PAF.txt. On this basis we have developed a SPARQL (http://www.w3.org/TR/sparql11-overview/) endpoint server for PRO using the open source edition of OpenLink Virtuoso (http://virtuoso.openlinksw.com). This allows our users to query against PRO data following the W3C SPARQL specification (http://www.w3.org/TR/sparql11-query/). A user can retrieve terms, subclass, and functional annotation from PRO, with or without inference. The PRO SPARQL endpoint is accessible from http://proconsortium.org/pro/pro_sparql.shtml. Query results are reported in a user-selectable common data exchange format. We have developed sample queries (shown on the web page referenced above) to guide our users in building their own queries. The queries include those that return either direct or all subclasses (7 or 85 terms, respectively) of TGF-β superfamily receptor type-1 (the latter without or with (180 terms) HermiT reasoner-based (http://www.hermit-reasoner.com) forward chaining), one that returns the functional properties of a PRO term, and one that takes advantage of a federated query to retrieve information from PRO and UniProtKB. Figure 4 presents the query and results of one of the samples.

## ACCESS TO PRO

PRO ontology files and associated tools (including additional graphical views (19,20)) are accessible at http://purl.obolibrary.org/obo/pr. The main PRO OBO file—connected to http://purl.obolibrary.org/obo/pr.obo—is now the reasoned version, pro_reasoned.obo; the non-reasoned version containing only single asserted parentage is pro.obo (available at ftp://ftp.proconsortium.org/databases/ontology/pro_obo). Both also come in OWL versions. Annotations are given in a separate file (PAF.txt), and a tab-delimited mapping file (promapping.txt) is also provided.

PRO terms are linked using the standard form http://purl.obolibrary.org/obo/PR_xxx, where xxx is either a nine-digit string or a UniProtKB accession. Users can request PRO terms or report issues via the PRO Tracker accessible from the home page. The Rapid Annotation interfaCE (RACE-PRO) (21) allows addition of experimental information to PRO terms.

PRO is distributed under the Creative Commons CC-BY 4.0 license (https://creativecommons.org/licenses/by/4.0/). Attribution can be made to the whole ontology using the original ontology URI (for example, http://purl.obolibrary.org/obo/pr.obo), or original term URIs for individual terms (as described in the previous paragraph). See http://obofoundry.org/ontology/pr.html for further details.
